# Anti-allergic rhinitis activity of α-lipoic acid via balancing Th17/Treg expression and enhancing Nrf2/HO-1 pathway signaling

**DOI:** 10.1038/s41598-020-69234-1

**Published:** 2020-07-27

**Authors:** Thi Van Nguyen, Chun Hua Piao, Yan Jing Fan, Dong-Uk Shin, Seung Yong Kim, Hyeon-Ji Song, Chang Ho Song, Hee Soon Shin, Ok Hee Chai

**Affiliations:** 1 Department of Anatomy, Jeonbuk National University Medical School, Jeonju, Jeonbuk 54896 Republic of Korea; 2 Division of Food Functionality Research, Korea Food Research Institute, 245, Nongsaengmyeong-ro, Iseo-myeon, Wanju-gun, Jeonbuk 55365 Republic of Korea; 3 Food Biotechnology Program, Korea University of Science and Technology, Daejeon, 34113 Republic of Korea; 4 Institute for Medical Sciences, Jeonbuk National University, Jeonju, Jeonbuk 54896 Republic of Korea; 5 Research Institute of Clinical Medicine of Jeonbuk National University-Biomedical Research Institute, Jeonbuk National University Hospital, Jeonju, 54907 Republic of Korea

**Keywords:** Allergy, Respiratory tract diseases

## Abstract

An ovalbumin (OVA)-induced allergic rhinitis (AR) mouse model was established to investigate whether α-Lipoic acid (LA) has a protective effect against upper respiratory tract inflammation. BALB/c mice were sensitized by intraperitoneal injection and challenged by intranasal application of OVA. Mice were orally administered various doses of LA once daily (2, 10, 50 mg/kg) and dexamethasone (Dex; 2.5 mg/kg) 1 h before OVA challenge. Allergic nasal symptoms, levels of OVA-specific immunoglobulins, cytokines, and transcription factors were measured. Nasal and lung histopathology were evaluated. LA administration significantly alleviated the nasal symptoms such as rubbing and sneezing, markedly reduced both serum OVA-specific IgE and IgG1 levels. The LA treatment group showed markedly up-regulated levels of the Treg cytokine IL-10 and Treg transcription factor Foxp3. In contrast, it showed down-regulated levels of the Th17 cytokine IL-17 and the Th17 transcription factor STAT3, and RORγ. LA greatly enhanced the nuclear factor erythroid-derived 2/heme oxygenase 1 (Nrf2/HO-1) pathway signaling and inhibited the activation of NF-κB/IκB, markedly suppressed the levels of pro-inflammatory cytokines TNF-α, IL-1β, IL-6, IL-8 and chemokine COX-2. The histologic alterations of nasal and lung tissues of AR mice were effectively ameliorated by LA. Based on these results, we suggest that LA could be a potential therapeutic agent in OVA-induced AR by virtue of its role in controlling the Th17/Treg balance and enhancing Nrf2/HO-1 pathway signaling.

## Introduction

Allergic rhinitis (AR) is one of the most common nasal conditions globally and is usually endured throughout life. The prevalence of AR has been estimated to be approximately 10–30% of the population worldwide^1^. AR is defined as an inflammatory disorder of the nasal mucosa induced by allergen exposure triggering IgE-mediated inflammation characterized by sneezing, nasal congestion, nasal itching, and rhinorrhea, in any combination^2^. Compared with other medical conditions, AR might not appear to be serious because AR itself is not life threatening. However, AR can cause emotional imbalance, sleeplessness, a significant economic burden, and severely decreased quality of life^3^. Most cases of AR respond to pharmacotherapy such as anti-histamines, intranasal steroids, and anti-leukotrienes. However, these clinical therapies can only alleviate the symptoms of AR and not modulate the pathophysiological basis in the early phase of AR. Therefore, it is necessary to focus on upstream regulatory factors of allergy to develop more effective management strategies for AR.

In the classical AR pathophysiology, elevated allergen-specific IgE level and imbalance of Th1/Th2 are known as the main immune deviation factors^4^. Recently, the imbalance of Treg/Th17 cells was also found to contribute to allergic airway inflammation^5^. Recent evidence has also revealed the roles of Th17 cells and their cytokines in promoting both eosinophilic and neutrophilic increase in the development of allergic disease^6^. In contrast, Treg cells play a central role in suppressing immune responses, sensing inflammation, and maintaining immune homeostasis^7^. In addition, Treg cells may be able to inhibit Th2/Th17 responses in allergic diseases^8^. Oxidative stress and their production of reactive oxygen species (ROS) were reported to associate with the development of several allergic inflammation diseases included AR^9^. ROS can elevate airway reactivity, increase mucosa permeability and mucus secretion, concurrently alter the expression of chemoattractant molecules to release inflammatory mediators^10^. Malondialdehyde (MDA) is an abundant individual aldehyde resulting from chain reactions of ROS and it was defined to be a biomarker of oxidative stress^11^. The Nrf2/HO1 signaling pathway has an important role against intracellular oxidative stress and inflammatory processing^12^. Nrf2 is an intracellular transcription factor that interacts with its downstream HO-1 protein to actuate its antioxidant effects. HO-1 has also pronounced to have anti-inflammatory by constraining pro-inflammatory cytokines IL-6 and TNF-a prodution^13^.

Alpha-lipoic acid (LA), an organic compound found in all human cells, has recently gained attention. LA is a vitamin-like antioxidant that acts as a free-radical scavenger. The human body produces LA naturally, but LA is also found in a variety of foods and as a dietary supplement. The antioxidant properties of LA have been linked to several benefits, including lower blood sugar levels, reduced inflammation, slowed skin aging, and improved nerve function^14^. It has also been described as a modulator of various inflammatory signaling pathways^15^. However, the role of LA in AR remains unknown. In the present study, we investigated the effect of LA treatment on allergic responses in an OVA-induced AR mouse model.

## Results

### LA treatment alleviated the nasal allergy symptoms in a dose-dependent manner

**Figure 1:**
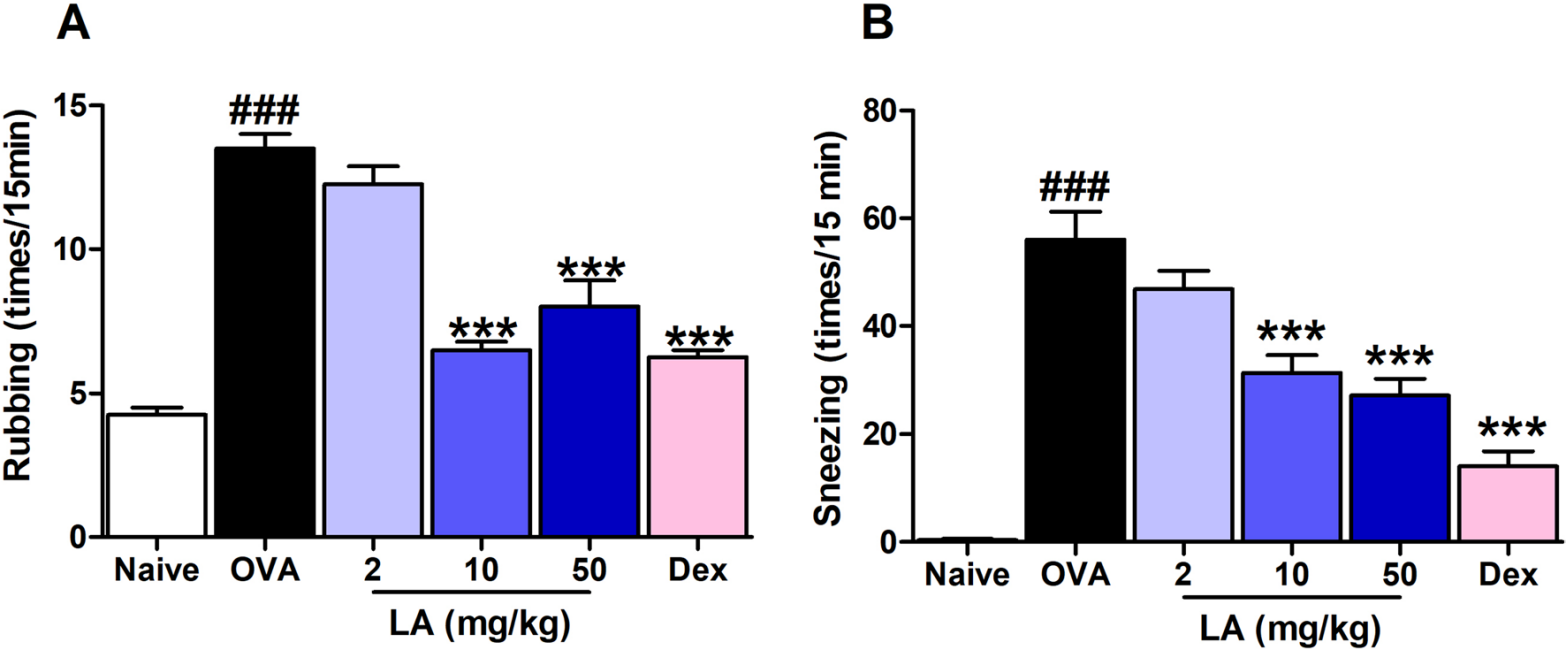
LA treatment alleviated AR nasal symptoms induced by OVA in a dose-dependent maner. (**A**) Rubbing. (**B**) Sneezing. The frequencies of rubbing and sneezing were recorded for 15 min after the last OVA intranasal challenge on d 27. Oral administration of LA 2, 10, 50 mg/kg and Dex 2.5 mg/kg significantly decreased the frequency of rubbing and sneezing time in AR mice. All results are shown as the mean ± SD (n = 6 per group). ^###^*P* < 0.001 versus Naive group. ***P < 0.001, vs OVA group. *AR* allergic rhinitis, *Dex* dexamethasone, *LA* lipoic acid.

To assess the effect of LA treatment on the early-phase allergic symptoms, the frequencies of rubbing and sneezing were determined for 15 min on the last day of OVA intranasal challenge. The frequencies of rubbing and sneezing after OVA challenge in OVA-induced AR mice were significantly higher than those in the Naive group. However, LA (2, 10, 50 mg/kg) administration significantly decreased the clinical allergic nasal symptoms compared to those in the OVA group in a dose-dependent manner. Similarly, the Dex (2.5 mg/kg) treatment also significantly alleviated the allergy symptoms in the AR mice model (Fig. 1A, B).

### LA treatment decreased infiltration of differential inflammatory cells in NALF in a dose-dependent manner

**Figure 2:**
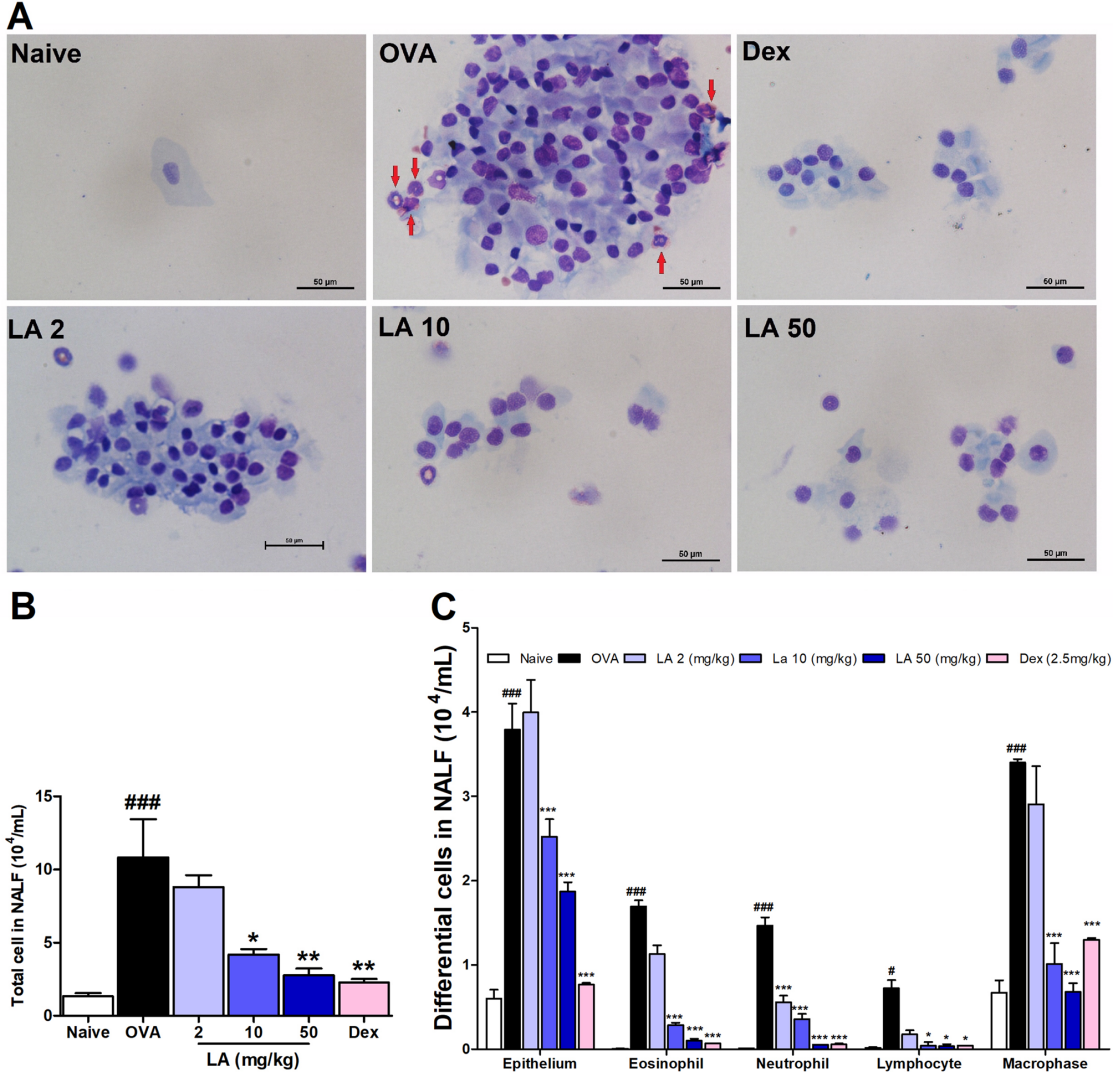
LA treatment reduced infiltration of differential inflammatory cells such as eosinophils in NALF in a dose-dependent manner. (**A**) Cytospin preparation (Diff-Quik staining, × 400). The number of (**B**) Total cells and (**C**) Differential cells. Oral administration of LA 10, 50 mg/kg and Dex 2.5 mg/kg notably suppressed the infitration of inflammatory cells in NALF of AR mice. Red arrows indicated eosinophil. All results are shown as the mean ± SD (n = 6 per group). ^#^*P* < 0.01, ^##^*P* < 0.01, ^###^*P* < 0.001, versus Naive group. *P < 0.05, **P < 0.01, ***P < 0.001, versus OVA group.

For each group, cytospin slides of NALF were stained with Diff-Quick stain kit to observe the differential cells by a microscope. In the OVA group, total cell numbers, and the differential cells including epithelial, eosinophils, neutrophils, lymphocytes and macrophages were markedly increased compared to those in the Naive group (Fig. 2A–C). In contrast, LA at doses of 10 and 50 mg/kg and Dex (2.5 mg/kg) treatments notably decreased the abundance of these inflammatory cells in NALF compared to that in the OVA group (Fig. 2A–C). The presence of differential inflammatory cells in NALF was determined by the Diff-Quik stain. Red arrows indicated the eosinophils. Eosinophils showed red-stained granules in cytoplasm and two lobes of their nucleus or sometimes appeared like a ring shape of nucleus. Neutrophils were appeared with pink cytoplasm and 2–5 lobes in their nucleus. Macrophages were large with a large dark blue nucleus that was usually bean shaped. Epithelial cell characterized by cilia, and its nucleus was located in opposite side with cilia.

### LA treatment suppressed allergic responses by regulating antigen-specific-immunoglobulins and declined the level of histamine in serum

**Figure 3:**
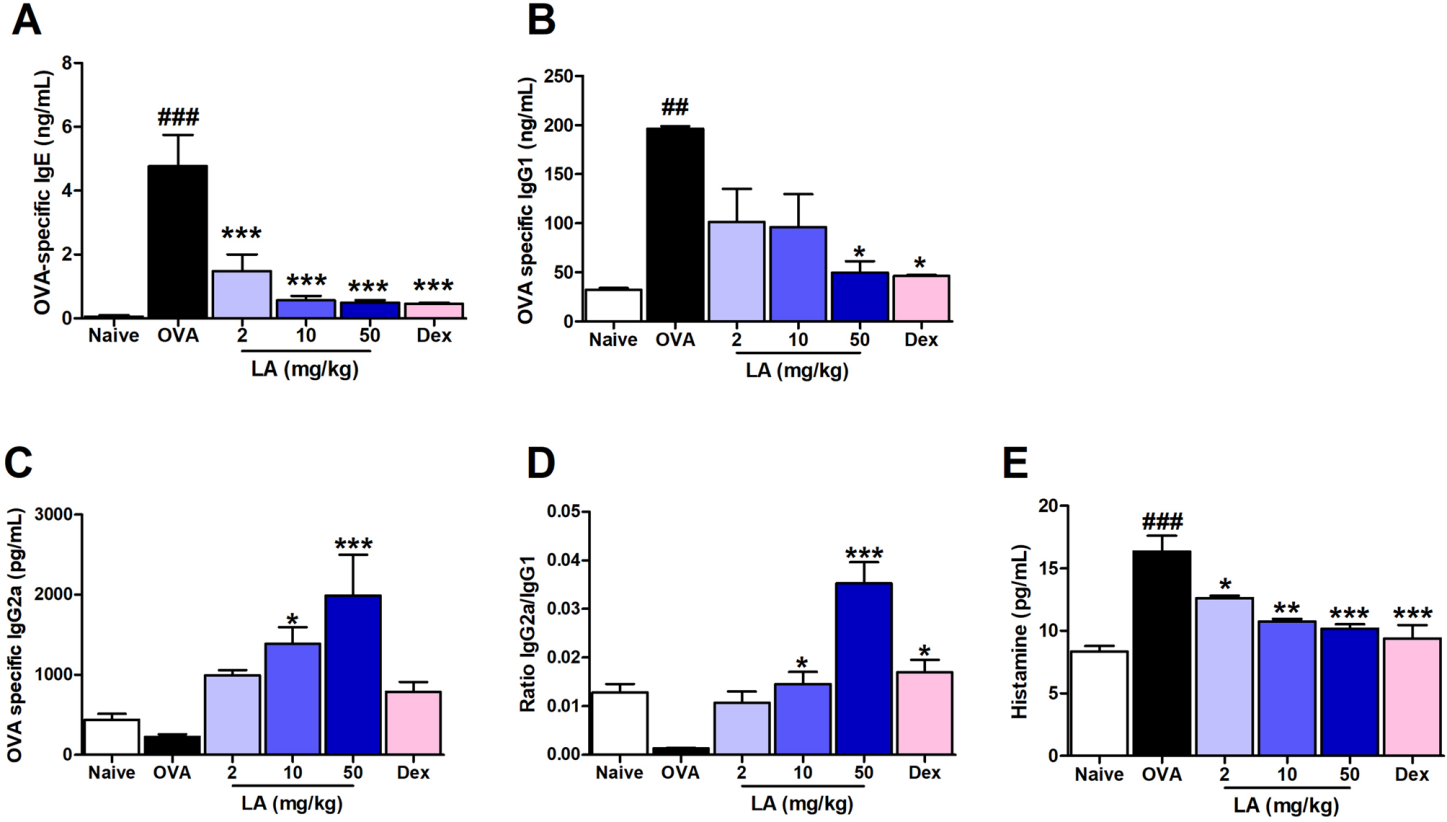
LA treatment suppressed allergic responses by regulating immunoglobulin levels in serum. (**A**) OVA-specific IgE, (**B**) OVA-specific IgG1, and (**C**) OVA-specific IgG2a in serum. Oral administration of LA 10, 50 mg/kg and Dex 2.5 mg/kg markedly down-regulated the level of OVA-specific IgE, IgG_1_ and up-regulated the level of IgG_2a_ in serum in AR mice. All results are shown as the mean ± SD (n = 6 per group). ^##^*P* < 0.01, ^###^*P* < 0.001, versus Naive group. *P < 0.05, ***P < 0.001, versus OVA group. *Ig* immunoglobulin.

Exposure to allergens, IgE-primed mast cell will be degranulated, released amount of histamine which start immediate allergic reactions. Repeated trigger to allergen stimulates more IgE and IgG1 production, which in turn strengthens the immune response. IgG_2a_ production is dependent on Th1 cells, which can regulate the activity of IgE and IgG_1_^16^. Therefore, we measured the production of anti-OVA IgE, IgG_1_, IgG_2a_ and histamine in serum using an ELISA kit. The results showed a significant up-regulation of anti-OVA IgE (Fig. 3A) and anti-OVA IgG_1_ (Fig. 3B) and there was a downward trend the level of anti-OVA-IgG_2a_ (Fig. 3C) in the OVA group compared with those in the Naive group. In contrast, LA administration dose-dependently resulted in markedly lower levels of anti-OVA IgE. At dose 50 mg/kg LA significantly decreased the level of anti-OVA IgG_1_. A considerably higher level of anti-OVA IgG2a serum compared with that in the OVA group was found in mice treated with 10 and 50 mg/kg LA, similar to that in Dex (2.5 mg/kg)-treated mice. The ratio of IgG_2a_ and IgG_1_ was greatly improved in the mice treated with LA 10, 50 mg/kg and Dex (Fig. 3D). The level of histamine also was found greatly increased in the OVA group compare with Naive group and it was significantly declined by the treatment with LA in a dose-dependent manner (Fig. 3E). These results showed that LA treatment suppressed the allergic responses via modulating the serum immunoglobulin subset and alleviated mast cell degranulation.

### LA treatment alleviated nasal mucosa thickness, accumulation of eosinophils and goblet cells, and hyperplasia in nasal tissue

**Figure 4:**
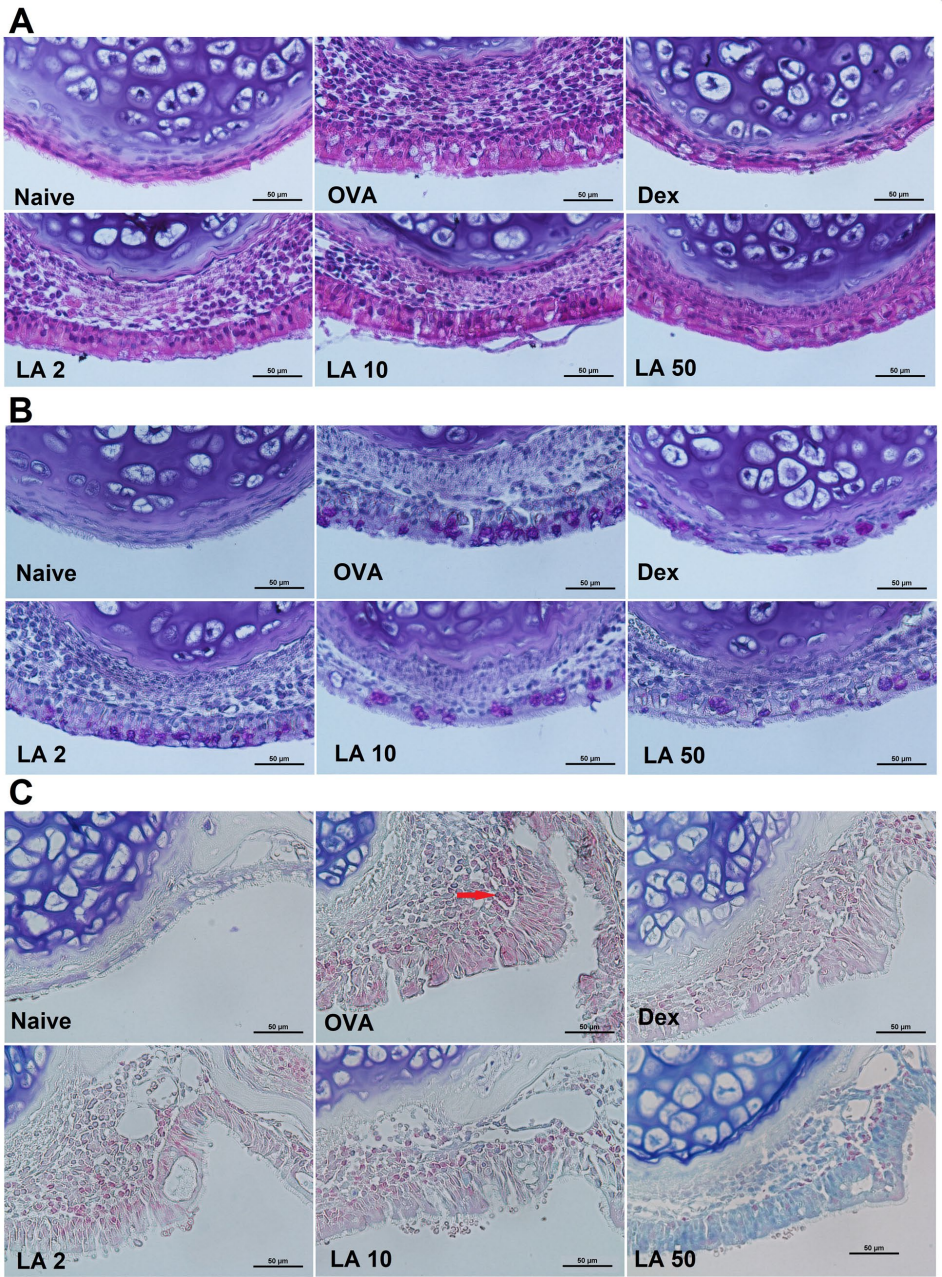
LA treatment alleviated nasal mucosa swelling and accumulation of infiltrated inflammatory cells and goblet cells. (**A**) H&E staining, (**B**) PAS staining, and (**C**) Giemsa staining. All pictures were at magnification of × 400. By H&E staining, the general structure of nasal tissue was observed. The subepithelium was significantly infiltrated by inflammation cells, the epithelium was swelling in OVA group, however, it was considerably reverted in treated group with LA and Dex. By PAS staining, the goblet cell was captured with violet color; by Giemsa staining, the eosinophil (indicated by red arrows) was screened with light red-stained cytoplasmic granules and dark purple nucleus with two lobles. The number of both goblet cell and eosinophil was markedly increased in OVA group and those were alleviated by LA and Dex.

H&E staining was performed to analyze the general morphology of the nasal cavity. Histological alterations were observed in the nasal mucosa of the OVA group; there was a major increase in the abundance of infiltrated inflammation cells in the subepithelium, which led to a significant increase in the mucosa thickness (Fig. 4A). The nasal mucosa was partially reverted to normal after LA administration. PAS staining revealed goblet cell hyperplasia in the nasal epithelium in the AR group compared to that in the Naive and LA treatment groups (Fig. 4B). The mucus hypersecretion with a violet color was highly conspicuous in the nasal mucosa epithelium of the OVA group, and it was alleviated in LA-treated mice. Nasal tissue staining with Giemsa revealed that treatment with LA and Dex strongly suppressed infiltration of eosinophil into the nasal mucosa; in the AR mice, the eosinophil count was increased (Fig. 4C). Eosinophils had red-stained cytoplasm, indicated by red arrows. Therefore, LA administration had a dose-independent protective effect on the nasal mucosa layer.

### LA treatment effectively suppressed inflammation in the lung tissue

**Figure 5:**
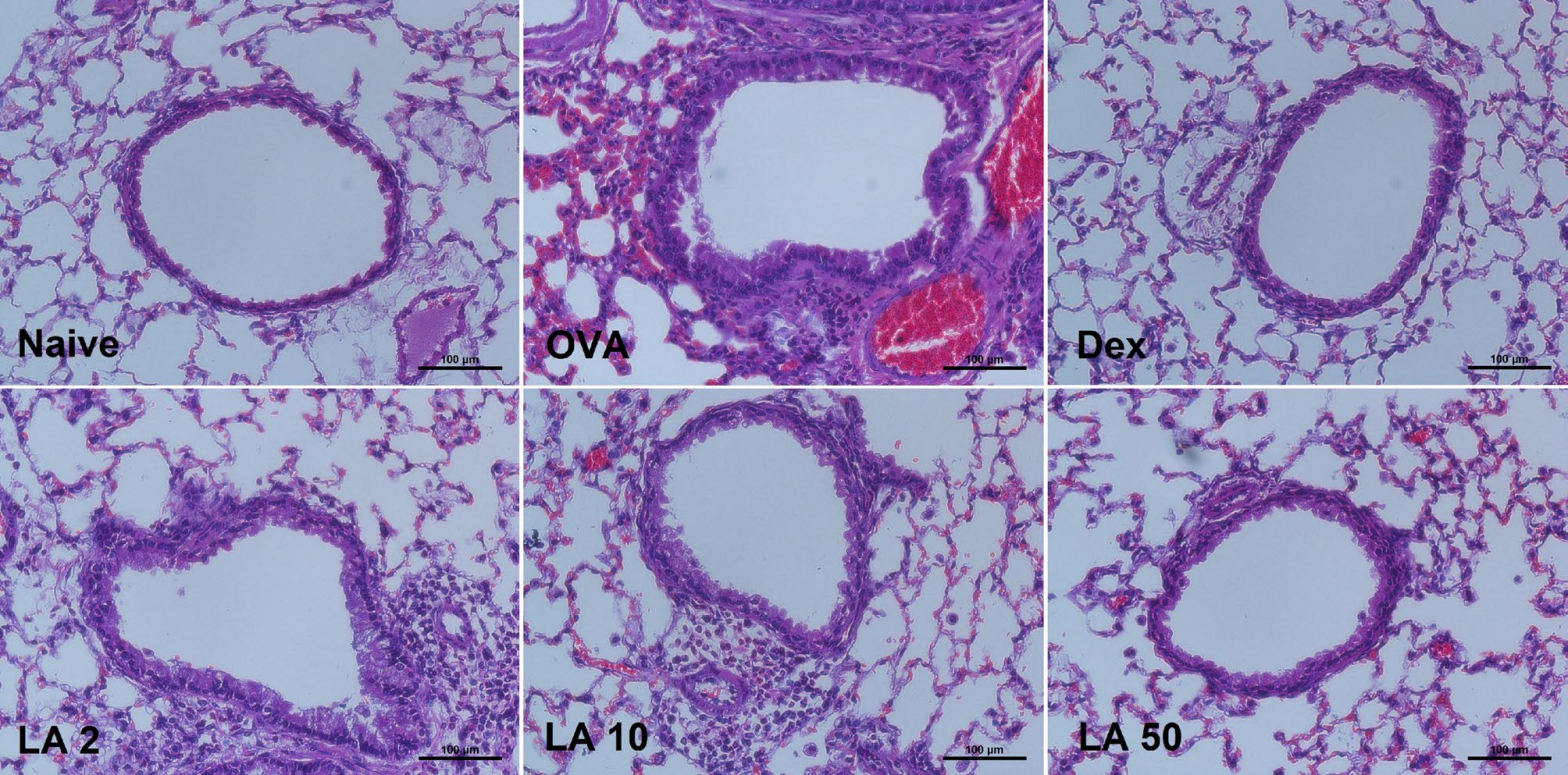
LA treatment alleviated inflammation in the lung tissue (H&E staining, × 200). Typical inflammation features were observed in the OVA group (including the infiltration of numerous cells around the bronchioles, alveoli, and blood vessels). The histology in treated group with LA 50 mg/kg and Dex was turned to be normal as naive group.

Inflammation in the upper-airway in AR may affect the lower-airway^17^. Therefore, H&E staining was performed to evaluate the histological change in the general structure of the lung tissue. Typical inflammation features were observed in the OVA group, including the infiltration of numerous cells around the bronchioles, alveoli, and blood vessels. However, mice treated with LA and Dex showed a marked decrease in inflammatory cell infiltration, and the LA 50 group’s lung tissues showed almost normal histology that was comparable to that of the naive group (Fig. 5).

### LA treatment up-regulated Treg and down-regulated Th17 cytokine production in NALF

**Figure 6:**
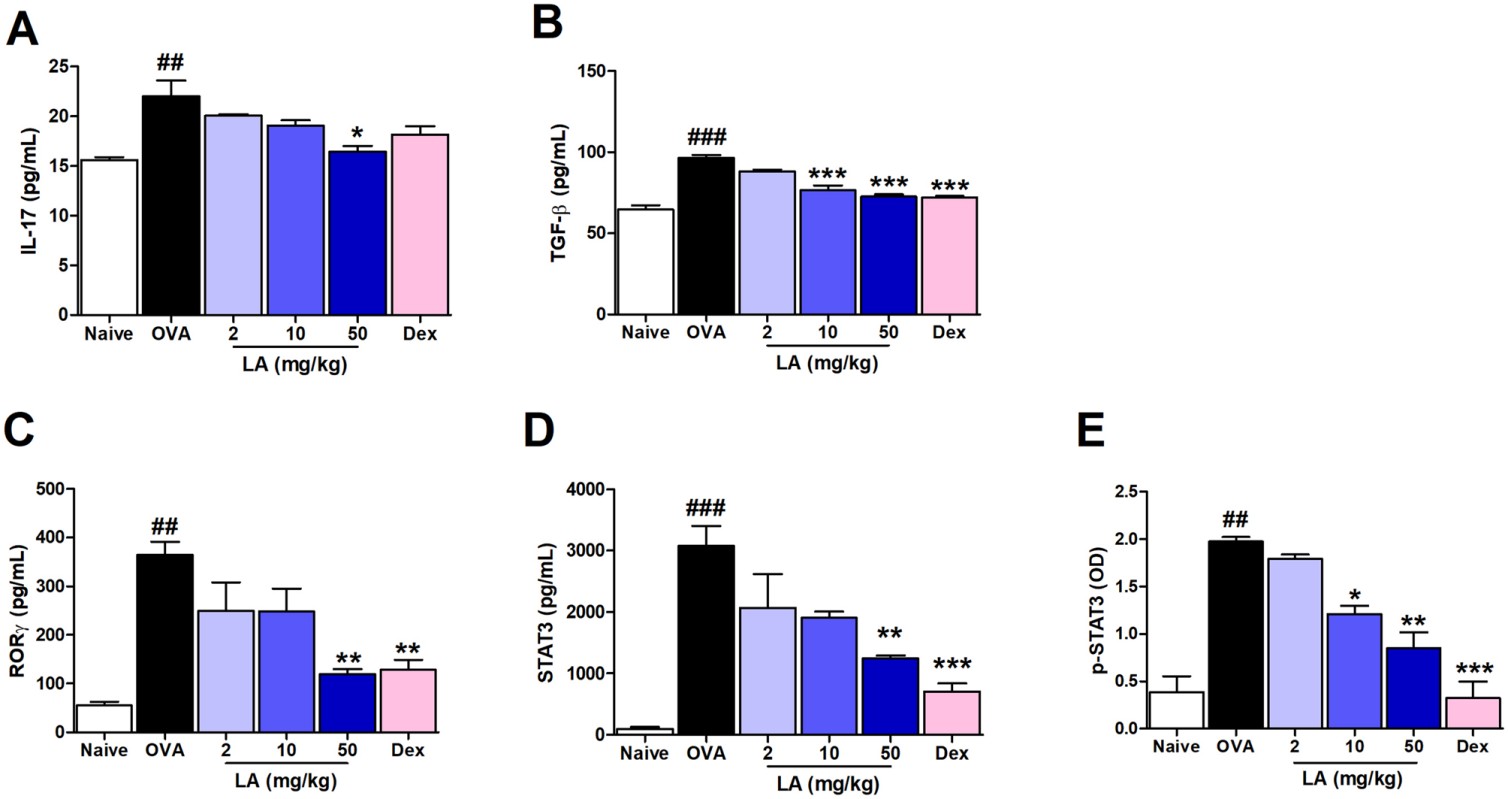
LA treatment down-regulated Th17 cytokine production and transcription factor levels in NALF. (**A**) IL-17, (**B**) TGF-β, (**C**) RORγ, (**D**) STAT3, and (**E**) p-STAT3. Oral administration of LA 50 mg/kg and Dex 2.5 mg/kg considerably decreased the cytokines and transcription fator belong to Th17 in AR mice. All results are shown as the mean ± SD (n = 6 per group). ^#^*P* < 0.01, ^###^*P* < 0.001, versus Naive group. *P < 0.05, **P < 0.01, ***P < 0.001, versus OVA group.

**Figure 7:**
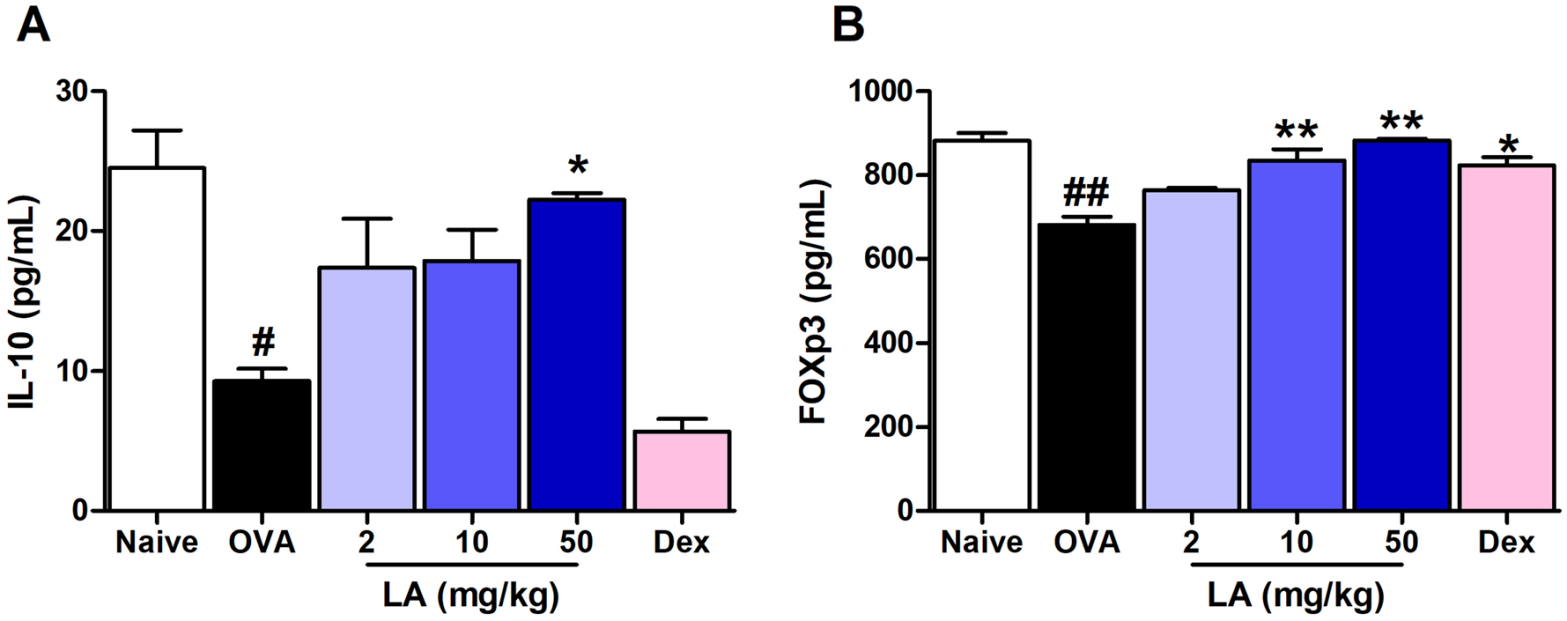
LA treatment markedly up-regulated Treg cytokine production. (**A**) IL-10 and (**B**) FOXp3.Oral administration of LA 50 mg/kg greatly improved the level of Treg cytokines in NALF of AR mice All results are shown as the mean ± SD (n = 6 per group). ^#^*P* < 0.01, ^##^*P* < 0.01, versus Naive group. *P < 0.05, **P < 0.01, ***P < 0.001, versus OVA group. *IL* interleukin, *FOXp3* Forkhead box P3.

Regulatory T cell and T helper 17 cells are two lymphocyte subsets with opposite actions: Th17 cells cause autoimmunity and inflammation, whereas Treg cells inhibit these phenomena and maintain immune homeostasis^18^. To investigate whether LA can positively interfere to protect the allergic mice, we checked the expression of Th17 and Treg cytokines in NALF. Figure 6A-E shows the significantly increase in the levels of the Th17 cytokines IL-17 and TGF-β, Th17 transcription factor RORγ, STAT3, and p-STAT3 in the OVA group compare with Naive group. However, the administration of LA at dose 50 mg/kg significantly alleviated those effects compared with that in the OVA group. The LA at dose 50 mg/kg treatment group also showed markedly up-regulated levels of the Treg cytokine IL-10. The level of Treg transcription factor Foxp3 in LA 10 and LA 50 group was notably higher than compared with the OVA group (Fig. 7A, B).

### LA treatment influenced on oxidative stress factors in NALF in a dose-dependent manner

**Figure 8:**
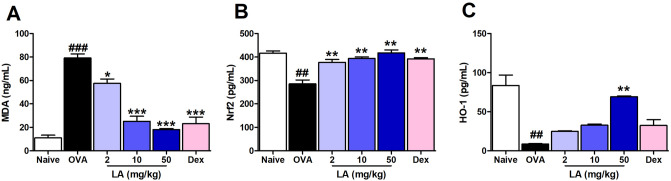
LA treatment influenced on oxidative stress factors in NALF in a dose-dependent manner. (**A**) MDA, (**B**) Nrf2 and (**C**) HO-1. Oral administration of LA 50 mg/kg significantly suppressed the level of a marker in oxidative stress MDA and activated Nrf2/HO-1 signaling by increasing the level of Nrf2 and HO-1 in NALF of AR mice. All results are shown as the mean ± SD (n = 6 per group). ^##^*P* < 0.01, ^###^*P* < 0.001, versus Naive group. *P < 0.05, **P < 0.01, ***P < 0.001, versus OVA group. *MDA* malondialdehyde, *Nrf2* nuclear factor erythroid 2-related factor 2, *HO-1* Heme oxygenase-1.

MDA and Nrf2/HO-1 axis are well known as the key elements in oxidative stress and anti-oxidant balance^19^. MDA is commonly described as an important biomarker of oxidative stress, and the Nrf2/HO-1 axis plays an essential role in defending against oxidative stress. Our study showed that the level of MDA was significantly increased in the OVA group compared with that in the control group, and it was sharply decreased by LA administration (Fig. 8A). In contrast, OVA-exposed mice had notably lower Nrf2 and HO-1 levels than the Naive group and LA (50 mg/kg)-treated mice had significantly elevated Nrf2 and HO-1 levels compared to the mice in the OVA group (Fig. 8B, C). There are vast of evidence reported that HO-1 has anti-inflammatory responsibility through inhibitting the activation of NF-kB^20^. To underline the anti-inflammatory effect of LA in the lung tissue via Nfr2/HO-1 signaling, we analyzed the levels of Nrf2, inflammatory factors including NF-kB, IkB and COX-2 in lung tissues using ProteinSimple capillary immunoassay (Wes) , a gel- and blot- free method. LA (10 mg/kg) inhibited inflammatory factors including COX-2 expression, NF-kB/IkB complex degradation, and NF-kB (p65) translocalization in lung tissues (Supplemental material Figure S2). Furthermore, LA (10 mg/kg) increased Nrf2 translocalization (Supplemental material Figure S2). These results showed that LA could suppress lung inflammation by activating Nrf2 similar to Fig. 8B.

### LA treatment notably reduced pro-inflammation cytokine levels in NALF and lung

**Figure 9:**
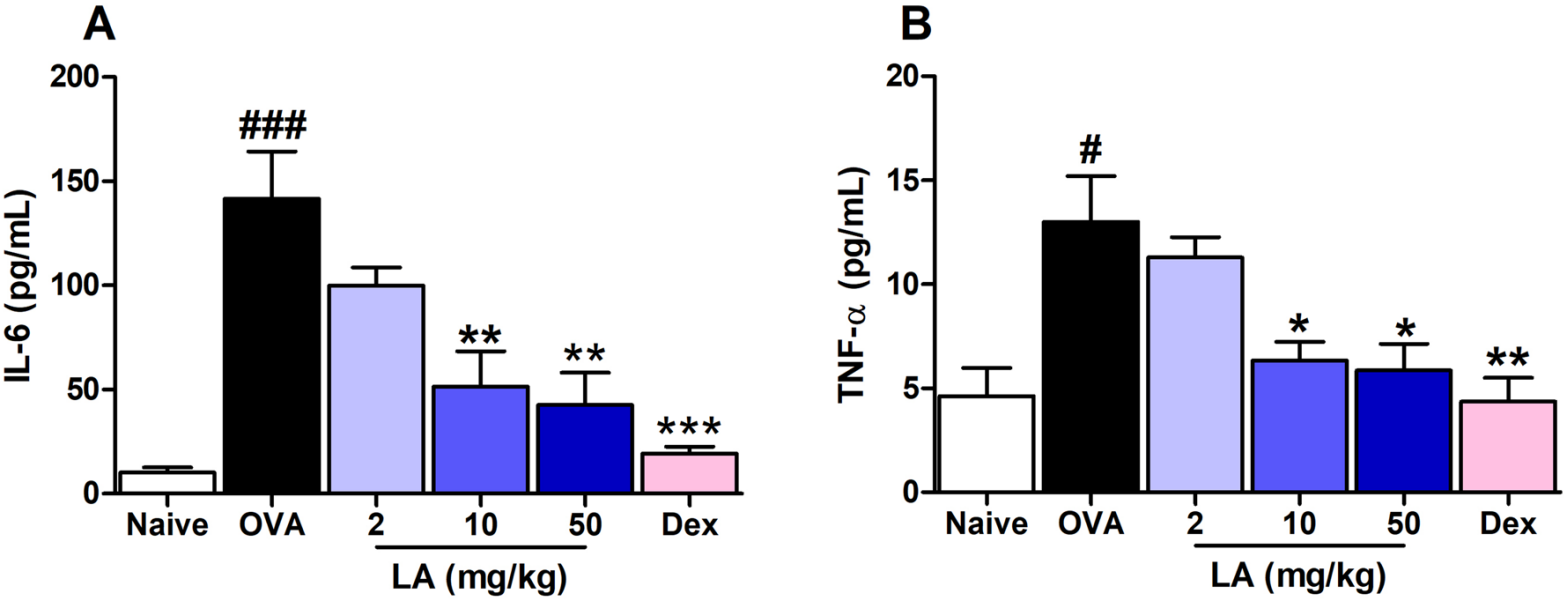
LA treatment reduced pro-inflammatory cytokine levels in NALF. (**A**) IL-6 and (**B**) TNF-α.Oral administration of LA 10, 50 mg/kg and Dex 2.5 mg/kg significantly decreased the level of both IL-6 and TNF-α in NALF of AR mice. All results are shown as the mean ± SD (n = 6 per group). ^#^*P* < 0.01, ^###^*P* < 0.001, versus Naive group. *P < 0.05, **P < 0.01, ***P < 0.001, versus OVA group. *IL* interleukin, *TNF-α* Tumor necrosis factor alpha.

TNF-α, similar to IL-6, is a pro-inflammatory cytokine, characterized by a broad spectrum of functions that are responsible for immune response activation^21^. We observed that the levels of both TNF-α and IL-6 were markedly increased in the OVA group compared with those in the Naive group. In addition, those levels were considerably reduced in the LA group compared with the levels in the OVA group (Fig. 9A, B). Also, we analyzed the mRNA levels of TNF-α, IL-1β, IL-6, IL-8 in lung tissue using PCR method. The mRNA levels of TNF-α, IL-1β, IL-6, IL-8 in lung tissue were markedly increased in the OVA group compared with those in the Naive group. However, LA (10 mg/kg) inhibited mRNA levels of TNF-α, IL-1β, IL-6, IL-8 in lung tissue (Supplemental material Figure S3). IL-1β (MIP-2 in mouse) and IL-8 correlated with IL-6 and TNF-α, resulting in similar trends. In other words, α-lipoic acid inhibited four factors (TNF-α, IL-1β, IL-6, IL-8) known as pro-inflammatory cytokines.

## Discussion

Repeated triggering of the allergic inflammatory response results in the IgE antibody binding to mast cells and basophils, leading to degranulation and release of preformed mediators such as histamine, leukotrienes, and prostaglandin D2^22^. This response causes increased vascular permeability, airway smooth muscle tightening, and enriched secretion of mucus^23^. These mediators also stimulate sensory nerves, which convey the sensations of nasal itch, congestion, rubbing, and sneezing within a minutes^24^. A strong augmentative correlation between antigen-specific IgE and IgG_1_ was reported in an allergic mouse model^25^. The presence of IgG_2a_ has been known as a marker for Th1; IgE and IgG_1_ have been known as markers for Th2 cells^26^. A higher IgG_2a_/IgG_1_ ratio is associated with protective immune responses in allergic inflammation diseases^27^. Therefore, in this study, we investigated whether LA can suppress allergic inflammation by preventing mast cell-IgE interaction and up-regulating the IgG_2a_/IgG_1_ ratio. Choi et al.^28^ reported that LA promotes the prevention or treatment of mast cell-mediated allergic diseases by inhibiting the release of histamine. In the present study also showed marked lower histamine in the serum of the AR mice treated with LA compares with the AR mice. It can be hypothesized that LA has mast cell degranulation prevention property. Additionally, in this study, the levels of OVA-specific IgE and IgG1 were significantly increased in the OVA group compared with the Naive group; however, oral administration with LA markedly decreased those compared with the OVA group. Moreover, the level of IgG_2a_ was considerably increased in the LA group compared with the OVA group (Fig. 3A–C). As a result, we also found that oral administration with LA decreased the nasal symptoms such as rubbing and sneezing (Fig. 1A, B). This implies that LA has an anti-allergic effect in the OVA-induced AR mouse model.

In the second phase of the AR response, as a result of cytokine or mediator release, the nasal mucosa is infiltrated with inflammatory cells such as basophils, eosinophils, neutrophils, mast cells, and mononuclear cells, which further maintain the nasal mucosal inflammatory reaction^29^. The present study demonstrated that the administration with LA clearly reduced the total cell number in NALF, especially the numbers of eosinophils and neutrophils. The H&E stain also showed a considerable increase in the infiltrated cells in the nasal mucosa in the OVA group, and this was significantly decreased in the groups treated with LA or Dex. Furthermore, the Giemsa stain results showed that the number of infiltrated eosinophils in the nasal mucosa was strongly decreased in LA and Dex groups (Fig. 4C). Previously, AR was described as a type of inflammation in the nose, but recent studies indicated that it may cause a systemic airway disease involving the entire respiratory tract^30^. The infiltrated inflammatory cells in the lung parenchyma were significantly higher in the AR group than in the control group. Therefore, it could confirm that asthma was concomitantly induced in the AR group. However, LA treatment markedly suppressed the infiltration of inflammatory cells in the lung tissue and bronchial contractions. According to these results, oral LA treatments could attenuate both upper and lower airway inflammation in the OVA-induced AR mouse model.

It is well-known that the imbalance of Th1/Th2 cell-mediated immunity has an important role in the pathogenesis of AR. Overexpression of Th2 cell-released cytokines including IL-4, IL-5, and IL-13 leads to stimulating B cells to produce allergen-specific IgE responsible for acute symptoms^31^. Recently, there is more evidence showing that Treg cells may promote and sustain tolerance to allergens by regulating both the innate and adaptive allergen-triggered immune response^32^. FOXP3, a master transcriptional regulator for Treg, is critical for the suppression of Th2 responses following exposure to allergens^33^. Valérie Dardalhon reported that FOXP3 can directly interact with GATA-3 (GATA Binding Protein 3) and inhibit its ability to transcribe genes encoded in Th2-related cytokines^33,34^. It was reported that LA decreased the level of Th2 production of IL-4 and IL-5 in an asthma mouse model^35^. In this study, we observed that the level of Foxp3 was sharply up-regulated in the LA-treated mouse group. This indicated that LA may alleviate rhinitis through suppressing the expression of Th2 cytokines via improving Treg Foxp3 production. Besides the level of Th1/Th2 cytokines, the imbalance in Th17/Treg is believed to lead to the development of various autoimmune and inflammatory diseases. Treg cells also exert their function by the release of anti-proinflammatory IL-10^36^. IL-10 potently reduces CD4^+^ T cell proliferation to inhibit the inflammatory cytokines production by lessening the presenting antigen^37^. Interestingly, TGF-β is well-known as a stimulating factor for both Treg and Th17 cells. Even the Naive CD4 T cell is the precursor of both Treg cells and Th17 cells, and TGF-β is necessary for the differentiation of Treg and Th17 cells^18^, however, Treg cells and Th17 play an opposite role in the inflammatory response. Th17 cells induce autoimmunity tissue injury and inflammation, whereas Treg cells inhibit autoimmunity and control undesired immune responses^38^. In the presence of pro-inflammatory IL-6, TGF-β induces Th17 cell differentiation from naïve T cells; however, in the absence of IL-6, naive T cells prioritize differentiation to Treg^18^. The up-regulation of IL-6 in plasma stimulated activation of STAT3, subsequently increasing expression of orphan nuclear receptor RORγ, a signature transcription factor required for differentiation of Th17 cells to their main cytokine IL-17^39^. In the present study, LA obviously suppressed the high level of IL-6 and TGF-β in the OVA group. Blocking the up-regulation of IL-6 leading to STAT3 signaling did not occur, resulting in the low level of RORγ and IL-17 in the NALF in the LA group. Moreover, LA significantly improved the levels of Treg-induced IL-10 and Foxp3. These results indicated that LA could alleviate rhinitis by balancing Treg cytokines and Th17 cytokines.

The NF-κB/IκB signaling strongly impacts on various aspects of innate and adaptive immune response in allergic inflammatory diseases like RA and asthma^40^. Regularly, inactivated NF-κB bounds to IκB to form a complex compound located in the cytoplasm^41^. IκB not only retains the level of NF-κB in the cytoplasm but also involves the reduction of NF-κB in the nucleus. Stress condition or the appearance of some pro-inflammatory cytokines IL-1β, TNF-α can activate NF-κB by inducing the phosphorylation of IκB^42^. The complex protein NF-κB/IκB will be degraded into two-part: phosphorated IκB and activated NF-κB. NFκB translocates to the nucleus, regulates expression of various pro-inflammatory genes that encode pro-inflammatory TNF-α, IL-1β, IL-6, IL-8, and chemokines cyclooxygenase-2 (COX-2)^43^. In this study, the level of IκB in the cytoplasm was significantly decreased in the OVA group compare with Naive group. These results can explain the majorly higher level of NFκB in the nucleus of AR mice compare with Naive mice. The level of COX-2 (supplementary Figure S2), the level of pro-inflammatory TNF-α, IL-1β, IL-6, IL-8 (supplementary Figure S3) also was found to be notably increased with the high level of NF-κB. Interestingly, LA at dose 10 mg/kg considerably increased IκB in cytoplasm lead to the decrease of those pro-inflammatory. According to these results, it could be emphasized that LA has an anti-inflammatory effect through inhibiting the activation of NF-κB /IκB signaling.

A number of researchers postulated that oxidative stress is an important mediator in the pathogenesis of allergic respiratory diseases such as asthma and rhinitis^44^. Oxidative stress occurs not because of exposure to environmental reactive oxygen species (ROS) such as air pollution and cigarette smoke but is also released as a byproduct of normal metabolic processes in all cells. Generally, our body maintains a balance between ROS and antioxidants. However, in allergic inflammation conditions, production of ROS from damaged cells or inflammatory reactions leads to an imbalance characterized by more oxidative stress^11^. MDA, which is known to be a hallmark of oxidative stress, was strongly produced in the AR group compared with the Naive group. In addition, the level of MDA was potently suppressed by LA (Fig. 8A). To defend against oxidative stress, Nrf2 is released from its complex protein Nrf2/Keap1 in the cytoplasm and then translocated into the nucleus where it regulates the expression of antioxidant and anti-inflammatory genes by binding to the antioxidant response element (ARE)^45^. The Nrf2-dependent anti-oxidant gene HO-1 is an anti-oxidant enzyme that plays an essential role in heme degradation to biliverdin, carbon oxide, and iron. In addition, HO-1 could inhibit the activation of NF-κB, directly block TNF-α and IL-6 inflammatory mediators and promote the production of anti-inflammatory cytokine IL-10^13^. Also, mice lacking Nrf2 exhibit an increased inflammatory response in the lung^46^. In this study, we found that LA notably improves the levels of Nrf2 in NALF and lung homogenate and the level of HO-1 in NALF compared with the AR group. This corresponds to the decrease in pro-inflammatory cytokines TNF-α, IL-1β, IL-6, IL-8, and chemokines cyclooxygenase-2 (COX-2) in the LA group compared with the OVA group. These results suggest that LA can protect the AR mouse against inflammation via the Nrf2/HO-1 signaling pathway.

In conclusion, we have shown that LA treatment has a positive effect against allergic inflammation in a mouse model of AR by improving Treg cell numbers and suppressing Th17 cell differentiation as well as through its antioxidant activity via the Nrf2/HO-1 signaling pathway. These obtained results suggested that LA treatment could be a promising strategy for the prevention and treatment of allergic airway diseases such as AR.

## Materials and methods

### Animals

Approximately 6-week-old male BALB/c mice (Damool Science, Dae-jeon, Korea) were used as experimental animals. The mice were housed under specific pathogen-free laboratory conditions at an ambient temperature of 23–25 °C with 50–60% relative humidity, and a 12 h diurnal light cycles. All experimental procedures followed the guidelines of the Institutional Animal Care and Use Committee of the Chonbuk National University Medical School (CBN 2019-071). The National Institutes of Health approved the study protocol.

### Establishment of AR model and treatment

BALB/c mice were randomly separated into six groups (n = 6): (1) Naive group, (2) OVA group, (3) LA 2 group, (4) LA 10 group, (5) LA 50 group, and (6) Dex group. The OVA-induced AR mouse model establishment and treatment protocol were described in Supplementary material Figure S1A. Briefly, OVA-induced AR mice were sensitized with 200 µL saline suspension including 50 μg OVA (Grade V, Sigma, St. Louis, MO, USA) and 25 µL of Imject Alum Adjuvant (Thermo Scientific, Rockford, MD, USA) to effectively stimulate the immune response for antibody production. The sensitization was performed 3 times on day 0, 7, and 14 by intraperitoneal injection. Next, mice were received treatment once daily with various doses of LA (T5625-1G, Sigma, St. Louis, MO, USA) (2, 10, or 50 mg/kg) and Dex (2.5 mg/kg) by oral administration for 13 days from day 15 to day 27. Mice in the OVA group were received saline. The sensitized mice were challenged by intranasal administration of 10 mg/mL OVA (20 µL in each nasal cavity) 1 h after receiving the treatment daily from day 21 to day 27. Mice were anesthetized by ether then sacrificed after the last OVA challenge 24 h^47^.

### Nasal symptoms

After the last OVA challenge, the numbers of nasal rubbing and sneezing times were counted for 15 min to evaluate early allergic response.

### Nasal lavage fluid (NALF) collection and analysis

After sacrifice, the trachea was opened, and 1 mL sterile saline solution was pumped gently into the nasal cavities by an 18-gauge catheter. NALF was collected from the anterior naris, centrifuged at 10,000 rpm for 10 min at 4 °C. The supernatant was taken to another tube then stored at − 80 °C for further determining cytokine levels. The cell pellets were resuspended in cold sterile saline. The total cell numbers were counted using a hemocytometer. Subsequently, 150 µL of NALF was benchtop centrifuged onto slides by the cytospin device (Centrifuge 5,403, Eppendorf, Hamburg, Germany) at 1,000 rpm for 10 min^48^. The relative numbers of different cell types including eosinophils, neutrophils, lymphocytes, macrophages, and epithelial cell numbers were determined by the Diff-Quik Staining kit (1-5-1 Wakinohama-Kaigandori, Chuo-Ku, Kobe, Japan). The inflammatory cells were counted at 400 × magnification under a light microscope (Leica, USA).

### Measurement of OVA-specific immunoglobulins and histamine in serum

Blood samples from each mouse were collected and immediately centrifuged for 10 min at 10,000 rpm, 4 °C. Serum layers were taken out separately and stored at − 80 °C prior to analysis. ELISA was performed on serum samples to detect OVA-specific IgE levels (439,807; BioLegend, Inc., San Diego, CA, USA); OVA-specific IgG_1_ (500,830; Cayman, MI, USA); OVA-specific IgG_2a_ (3,015; Chondrex, Inc., Washington, USA), and histamine (ab213975; Abcam, United Kingdom) according to the manufacturer’s instructions. Absorbance was measured using the Bio-Rad 680 microplate reader (Bio-Rad Laboratories, Inc., Hercules, CA, USA) at 450 nm^48^.

### Histological examination

For nasal and lung histopathology examination, the head and lung tissues were placed in a liquid fixing agent (10% neutral buffered formalin) for 3 days at 23 ± 2 °C. Then, the samples were dehydrated with a gradually increasing concentration series of ethanol, cleared the ethanol by xylene, and then embedded in paraffin. The head tissues were decalcified in a Calci Clear-Rapid (National Diagnostics) solution for 2 day at room temperature (20–25 °C) before the dehydrating process. The tissues were sectioned at 4.5 µm thickness. Hematoxylin and eosin (H&E) (Sigma, St. Louis, MO) staining was performed to estimate general morphology; Giemsa (Sigma, St. Louis, MO) and periodic acid-Schiff (PAS) (Sigma, St. Louis, MO) staining were used to observe eosinophil accumulation and goblet cell hyperplasia^49^.

### Quantification of cytokines by ELISA

The supernatant was collected from the NALF for quantitating cytokine release. Cytokine quantitation kits were used to measure cytokine levels including those of IL-10, IL-17, IL-6, TNF-α (R&D Systems, Inc., Minneapolis, USA); Nrf-2, HO-1, MDA, Foxp-3 (BD Biosciences, San Diego, CA, USA); STAT3, p-STAT3 (Invitrogen, Carlsbad, California); and RORγ (Fine Biotech, Wuhan, Hubei, China). All assays were performed in duplicate, standard solutions and NALF samples were transferred to 96-well plate which was prior coated with a target cytokine capture antibody, incubated for 2 h at room temperature, then washed for 4 times. After incubated 2 h with an appropriate biotin-conjugated antibody, the wells were aspirated and washed again 4 times, and then a horseradish peroxidase (HRP) was added to each well. Removing the excess HRP conjugate by washing, a substrate solution was added to convert the solution to a detectable form. Add stop solution to each well, read the optical density of each well at 450 nm immediately^47,48^.

### Measurement of pro-inflammatory cytokines by RT-PCR

Lung tissues were isolated from allergic rhinitis mice model and homogenized for mRNA extraction using lung dissociation kit (Miltenyi biotec, Bergisch Gladbach, Germany). Total RNA was recovered and purified using an RNeasy Mini Kit (Qiagen, Germany) according to the manufacturer’s instructions. cDNA was synthesized using a QuantiTect Reverse Transcription Kit (Qiagen, Germany). First-strand cDNA was prepared from 1 μg of total RNA. The samples were subjected to real-time PCR using SYBR Green master mix on a Rotor-Gene Q 2plex System (Qiagen, Germany). The gene expression levels were normalized to those of β-actin. Relative gene expression changes were calculated using the 2-delta CT method and reported as fold change over the control samples. The sequence of primers was as follows: IL-8, (forward) 5′-CGG CAA TGA AGC TTC TGT AT-3′ and (reverse) 5′-CCT TGA AAC TCT TTG CCT CA-3′; IL-1β, (forward) 5′-ACCTGGGCTGTCCTGATGAGAG-3′ and (reverse) 5′-GTT GAT GTG CTG CTG CGA GAT-3′; TNF-α, (forward) 5′-TCT TCT CAT TCC TGC TTG TGG-3′ and (reverse) 5′-GGT CTG GGG CAT AGA ACT GA-3′; IL-6, (forward) 5′-TGG GAC TGA TGC TGG TGA CAA C-3′ and (reverse) 5′-AGC CTC CGA CTT GTG AAG TGG T-3′; β-actin, (forward) 5′-GCT CAG TAA CAG TCC GCC TAG A-3′ and (reverse) 5′-TGT CCA CCT TCC AGC AGA TGT-3′. The primers used in this experiment were purchased from Bioneer (Daejeon, Korea).

### Protein simple capillary immunoassay (Wes)

Lung tissues were isolated from allergic rhinitis mice model and homogenized for proteins extraction using lung dissociation kit (Miltenyi biotec, Bergisch Gladbach, Germany). The extracts were fractionated to nuclear and cytoplasmic fractions by kit (Thermo Fisher scientific, Waltham, MA, USA). COX-2 expression (primary antibody 1:50) and IκB degradation (primary antibody 1:50) were measured in cytoplasmic fraction, and translocalization of NF-κB (p65) (primary antibody 1:50) and NRF2 (primary antibody 1:50) were detected in nuclear fraction. Expression of proteins in each fraction was quantitatively analyzed by ProteinSimple capillary immunoassay WES system (Protein simple, San Jose, CA, USA).

### Statistical analysis

The experimental data were analyzed using GraphPad Prism 6.0 software (v5.0, La Jolla, CA, USA). Two-way ANOVA followed by Bonferroni’s Multiple Comparison was used to compare the result of differential cells in NALF between groups. Statistical analyses of the other data were analyzed using one-way ANOVA followed by Turkey’s test. Values for all measurements are expressed as means ± standard error (SE) of independent experiments. *P* < 0.05 was considered to indicate the statistically significant^49^.

## Supplementary information

Supplementary Information 1.

## Abbreviations

AR: Allergic rhinitis
Dex: Dexamethasone
Foxp3: Factor forkhead box P3
HO-1: Heme oxygenase-1
Ig: Immunoglobulin
IL: Interleukin
LA: α-Lipoic acid
MDA: Malondiadehyde
NALF: Nasal lavage fluid
Nrf2: Nuclear factor (erythroid-derived 2)-like 2
OVA: Ovalbumin
RORγ: RAR-related orphan receptor gamma
ROS: Reactive oxygen species
STAT3: Signal transducer activator of transcription 3
TGF-β: Transforming growth factor β
TNF-α: Tumor necrosis factor alpha
Treg: T regular cell
