# Supplementary material for: Anti-allergic rhinitis activity of α-lipoic acid via balancing Th17/Treg expression and enhancing Nrf2/HO-1 pathway signaling
Source: Sci Rep. 2020 Jul 27;10:12528. doi: 10.1038/s41598-020-69234-1 (PMC7385155; doi:10.1038/s41598-020-69234-1)

Supplementary material for

Anti-allergic rhinitis activity of α-lipoic acid via balancing Th17/Treg expression and enhancing Nrf2/HO-1 pathway signaling

Thi Van Nguyen[a], Chun Hua Piao[a], Yan Jing Fan[a], Dong-Uk Shin[b,c], Seung Yong Kim[b], Hyeon-Ji Song[b], Chang Ho Song[a,d], Hee Soon Shin[b,c], Ok Hee Chai[a,d,e]*

[a] Department of Anatomy, Jeonbuk National University Medical School, Jeonju, Jeonbuk, 54896, Republic of Korea.

[b] Division of Food Functionality Research, Korea Food Research Institute, 245, Nongsaengmyeong-ro, Iseo-myeon, Wanju-gun, Jeonbuk 55365, Republic of Korea.

[c] Food Biotechnology Program, Korea University of Science and Technology, Daejeon 34113, Republic of Korea.

[d] Institute for Medical Sciences, Jeonbuk National University, Jeonju, Jeonbuk, 54896, Republic of Korea.

[e] Research Institute of Clinical Medicine of Jeonbuk National University- Biomedical Research Institute of Jeonbuk National University Hospital, Jeonju, 54907, Republic of Korea.

Figure S1. Experimental protocol for establishing the murine allergic rhinitis (AR) model and treatment with LA (A) and the structure of LA (B). Mice were sensitized (I.p) 3 times on day 0, 7, and 14, and challenged (I.n) from day 21 to day 27 by OVA. Mice in the OVA group were sensitized and challenged by OVA. Mice in LA 2, LA 10, LA 50, or dexamethasone (Dex) treatment groups were sensitized and challenged by OVA and administered 2, 10, or 50 mg/kg LA (once a day by oral gavage; 200 µL) or 2.5 mg/kg Dex from day 15 to day 27 for 13 days. The mice in the Naive group were not sensitized, challenged, and treated. The mice were sacrificed on day 28.

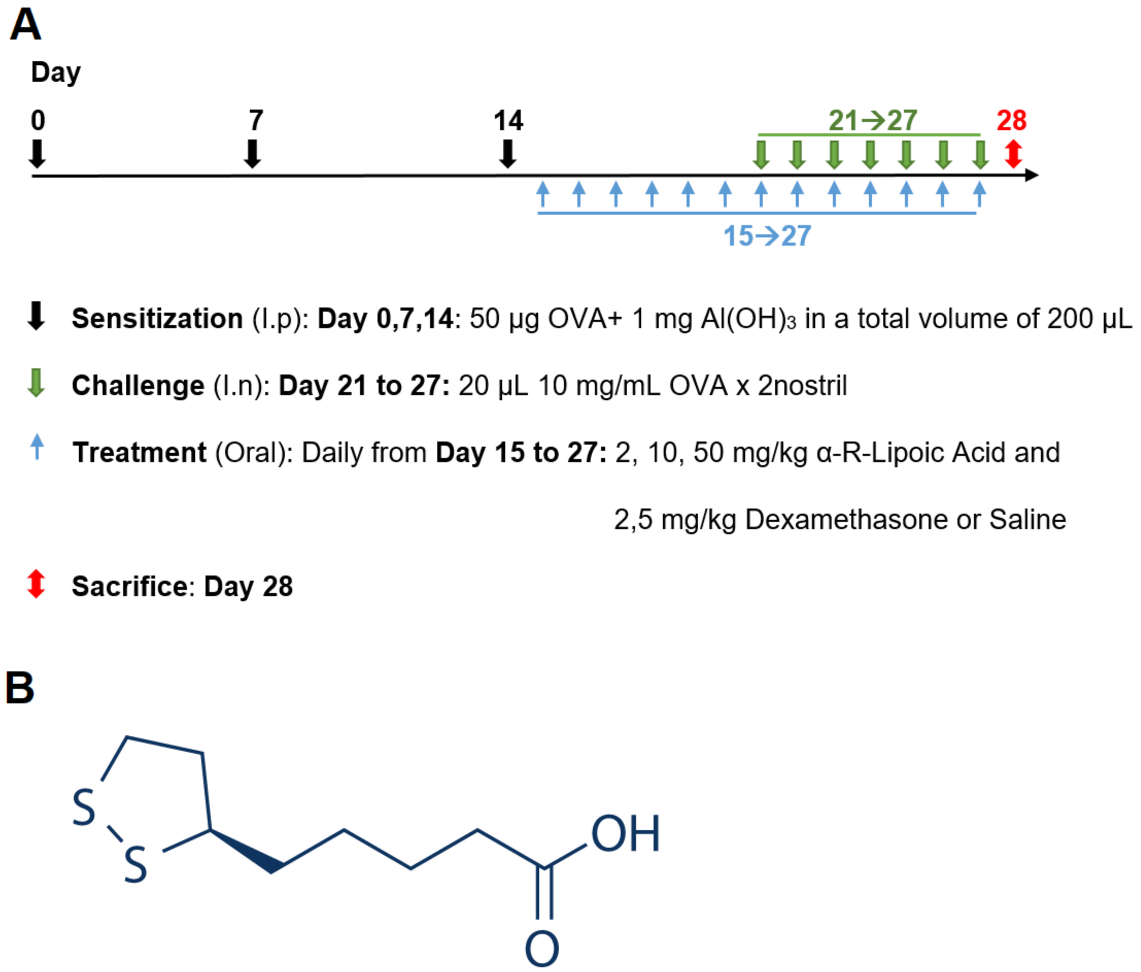

Figure S2. A. Anti-inflammatory effect of α-lipoic acid on inflammatory factors in lung tissues. Lung tissues were isolated from allergic rhinitis mice model and homogenized for proteins extraction using lung dissociation kit (Miltenyi biotec, Bergisch Gladbach, Germany). The extracts were fractionated to nuclear and cytoplasmic fractions by kit (Thermo Fisher scientific, Waltham, MA, USA). COX-2 expression (primary antibody 1:50) and IkB degradation (primary antibody 1:50) were measured in cytoplasmic fraction, and translocalization of NK-kB(p65) (primary antibody 1:50) and NRF2 (primary antibody 1:50) were detected in nuclear fraction. Expression of proteins in each fraction was quantitatively analyzed by ProteinSimple capillary immunoassay WES system (Protein simple, San Jose, CA, USA). **B. Full length gels and blots of inflammatory factors in lung tissues**

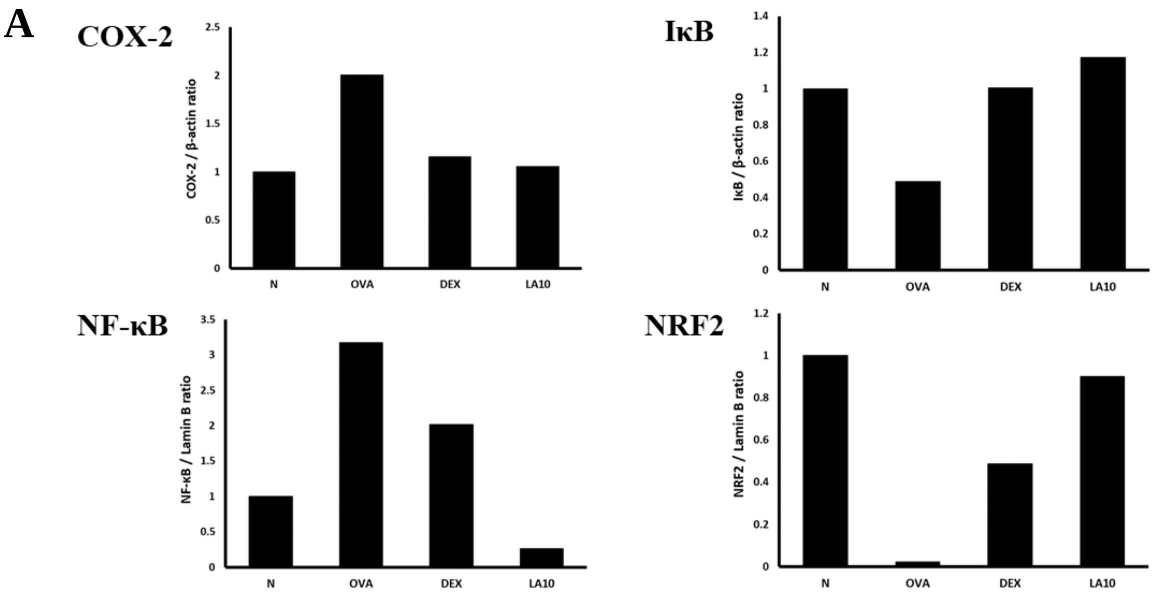

B

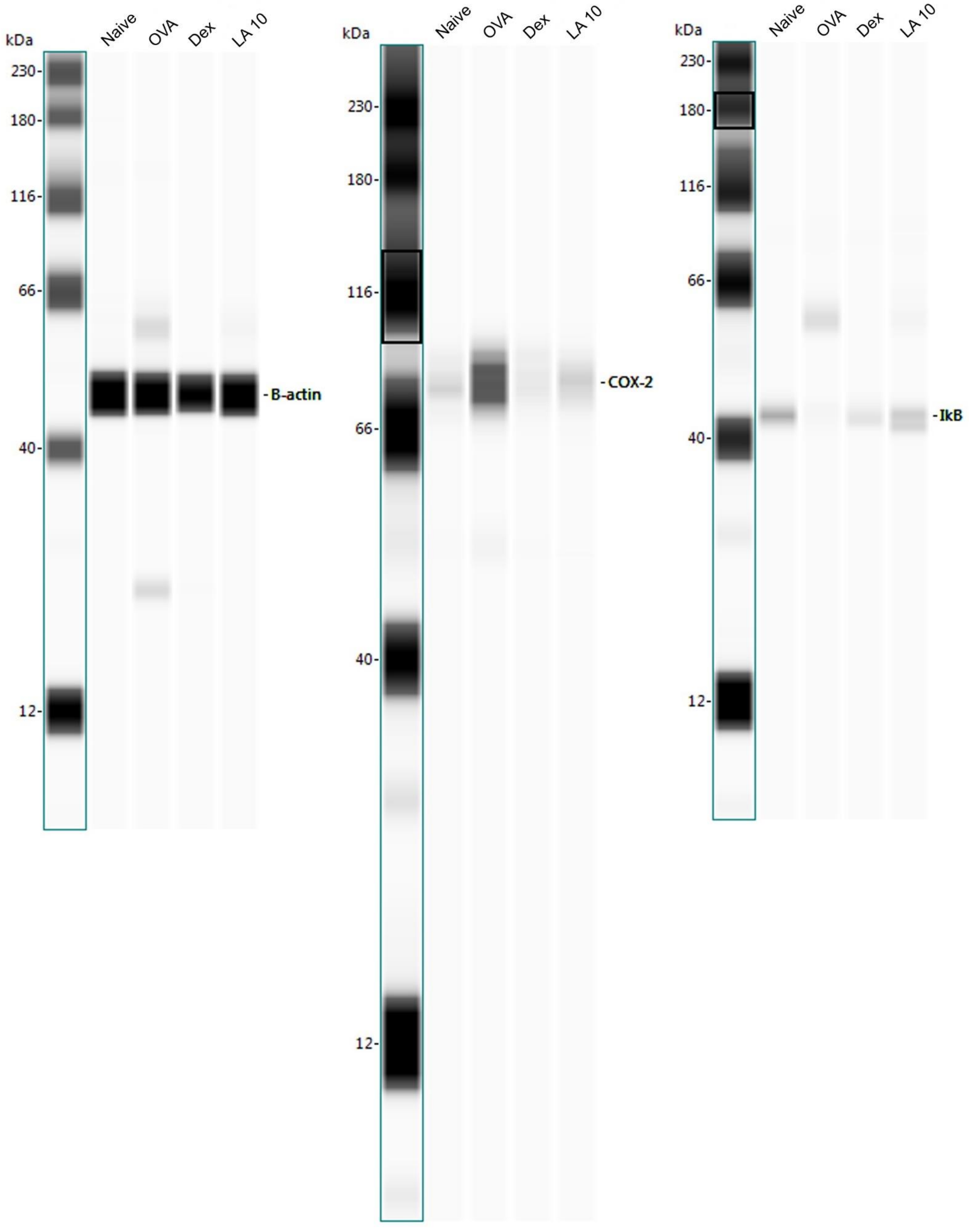

kDa
Naive
OVA
Dex
LA 10
230
180
116
66
40
12
B-actin
kDa
Naive
OVA
Dex
LA 10
230
180
116
66
40
12
COX-2
kDa
Naive
OVA
Dex
LA 10
230
180
116
66
40
12
IkB

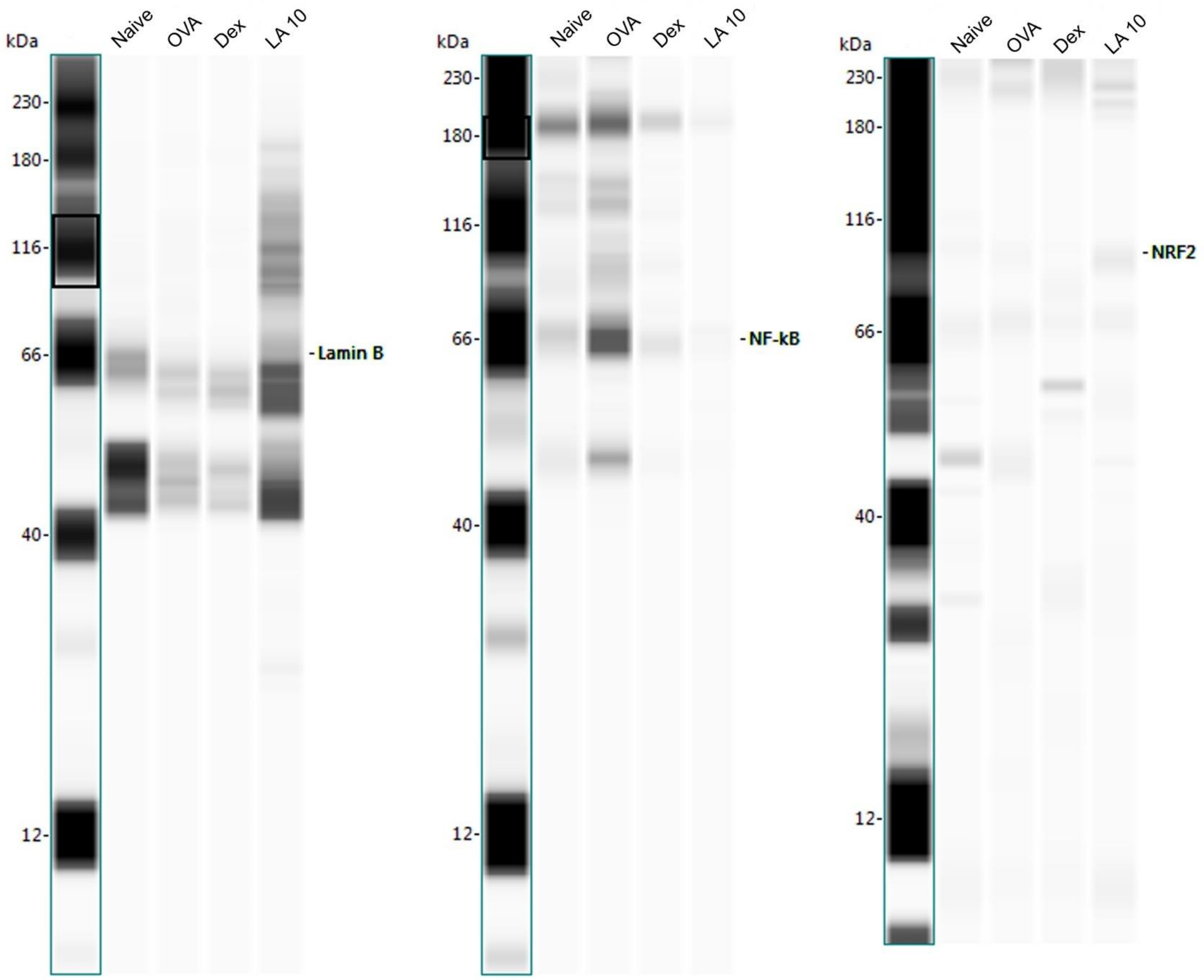
kDa
Naive
OVA
Dex
LA 10
230
180
116
66
40
12
Lamin B
NF-kB
NRF2

Figure S3. **Anti-inflammatory effect of α-lipoic acid on inflammatory cytokines in lung tissues.** Lung tissues were isolated from allergic rhinitis mice model and homogenized for mRNA extraction using lung dissociation kit (Miltenyi biotec, Bergisch Gladbach, Germany). Total RNA was recovered and purified using an RNeasy Mini Kit (Qiagen, Germany) according to the manufacturer's instructions. cDNA was synthesized using a QuantiTect Reverse Transcription Kit (Qiagen, Germany). First-strand cDNA was prepared from 1 μg of total RNA. The samples were subjected to real-time PCR using SYBR Green master mix on a Rotor-Gene Q 2plex System (Qiagen, Germany). The gene expression levels were normalized to those of β-actin. Relative gene expression changes were calculated using the 2-delta CT method and reported as fold change over the control samples. The sequence of primers was as follows: IL-8, (forward) 5'-CGG CAA TGA AGC TTC TGT AT-3' and (reverse) 5'-CCT TGA AAC TCT TTG CCT CA-3'; IL-1β, (forward) 5'-ACCTGGGCTGTCCTGATGAGAG-3' and (reverse) 5'-GTT GAT GTG CTG CTG CGA GAT-3'; TNF-α, (forward) 5'- TCT TCT CAT TCC TGC TTG TGG-3' and (reverse) 5'-GGT CTG GGG CAT AGA ACT GA-3'; IL-6, (forward) 5'- TGG GAC TGA TGC TGG TGA CAA C- 3' and (reverse) 5'- AGC CTC CGA CTT GTG AAG TGG T- 3'; β-actin, (forward) 5'-GCT CAG TAA CAG TCC GCC TAG A-3' and (reverse) 5'-TGT CCA CCT TCC AGC AGA TGT-3'. The primers used in this experiment were purchased from Bioneer (Daejeon, Korea).

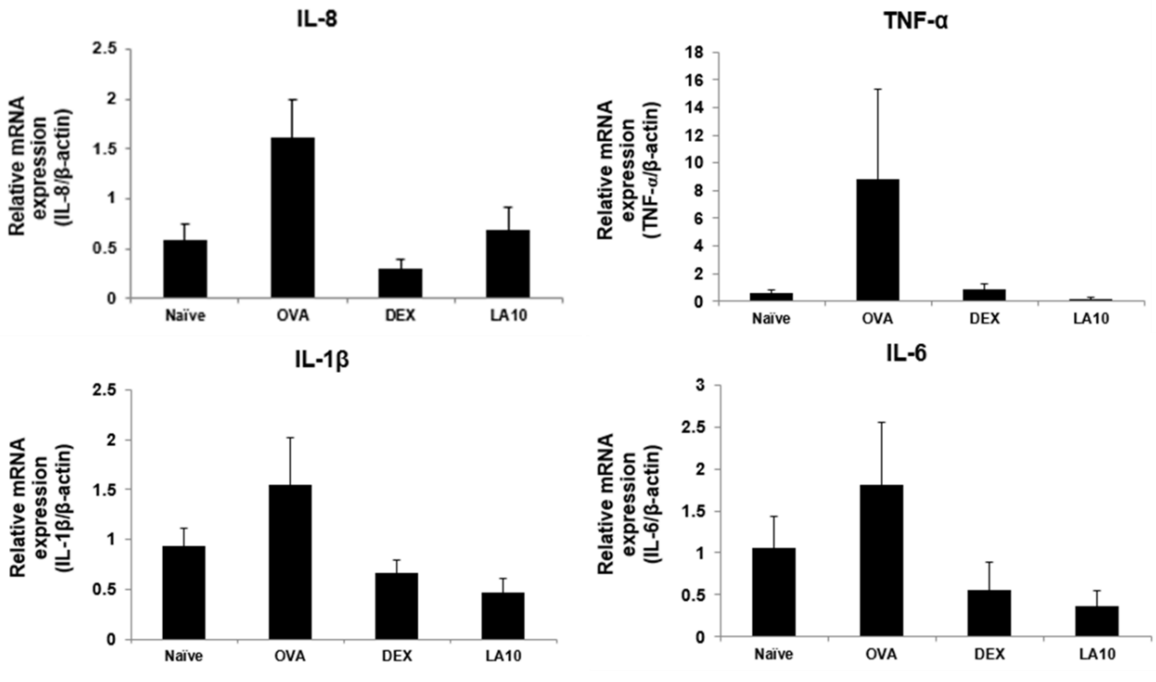

Supplement: Supplementary file 1 — Supplementary Information 1. [file 41598_2020_69234_MOESM1_ESM.pdf]
